# Biological Rationale for Targeting MEK/ERK Pathways in Anti-Cancer Therapy and to Potentiate Tumour Responses to Radiation

**DOI:** 10.3390/ijms20102530

**Published:** 2019-05-23

**Authors:** Francesco Marampon, Carmela Ciccarelli, Bianca Maria Zani

**Affiliations:** 1 Dipartimento di Scienze Radiologiche, Oncologiche e Anatomo-Patologiche della Sapienza, Università di Roma, Viale Regina Elena, 324, 00161 Roma, Italy; francesco.marampon@uniroma1.it; 2 Dipartimento di Scienze Cliniche Applicate e Biotecnologiche, Università degli Studi dell’Aquila, Coppito II Via Vetoio, 67100 L’Aquila, Italy; Carmela.ciccarelli@univaq.it

**Keywords:** MAPK signalling, ERK1/2 splicing isoforms, ERK2 mutants, MAPK inhibitor, chemo- and radio-resistance

## Abstract

ERK1 and ERK2 (ERKs), two extracellular regulated kinases (ERK1/2), are evolutionary-conserved and ubiquitous serine-threonine kinases involved in regulating cell signalling in normal and pathological tissues. The expression levels of these kinases are almost always different, with ERK2 being the more prominent. ERK1/2 activation is fundamental for the development and progression of cancer. Since their discovery, much research has been dedicated to their role in mitogen-activated protein kinases (MAPK) pathway signalling and in their activation by mitogens and mutated RAF or RAS in cancer cells. In order to gain a better understanding of the role of ERK1/2 in MAPK pathway signalling, many studies have been aimed at characterizing ERK1/2 splicing isoforms, mutants, substrates and partners. In this review, we highlight the differences between ERK1 and ERK2 without completely discarding the hypothesis that ERK1 and ERK2 exhibit functional redundancy. The main goal of this review is to shed light on the role of ERK1/2 in targeted therapy and radiotherapy and highlight the importance of identifying ERK inhibitors that may overcome acquired resistance. This is a highly relevant therapeutic issue that needs to be addressed to combat tumours that rely on constitutively active RAF and RAS mutants and the MAPK pathway.

## 1. Introduction

Extracellular signal-regulated kinase (ERK1/2) belongs to the mitogen-activated protein kinases (MAPK) family of kinases, comprising p38 and c-Jun NH2-terminal kinase (JNK, also known as stress-activated protein kinase, or SAPK). These pathways respond to various extracellular stimuli and control cell cycle progression, proliferation, cytokinesis, transcription, differentiation, senescence, cell death, migration, GAP junction formation, actin and microtubule networks, neurite extension and cell adhesion motility, stress response, survival and apoptosis [1]. As a result, impairments in MAPK signalling pathways are reported in a range of human diseases, including cancer and neurodegenerative disorders such as Alzheimer’s disease, Parkinson’s disease and amyotrophic lateral sclerosis [2].

The MAPK cascades consist of a family of evolutionary-conserved, signalling proteins represented by three core kinases, namely MAPK3K, MAPKK and MAPK. The MAPK cascade works through sequential phosphorylations: MAP3Ks phosphorylate and activate MAP2Ks, which in turn phosphorylate and activate MAPKs [3].

These events occur through sequential interactions between the kinase components or through the help of scaffold proteins that enable the activation of kinases in the multiple complexes [4]. Moreover, each MAPK component plays a role that is unrelated to the classical MAPK kinase cascade [5].

Extracellular signal-regulated kinases (ERK-1 and -2; MAPK-3 and -1) were the first mammalian MAPKs to be identified. ERKs are well known as insulin and mitogen-activated MAPKs, which engage the RAS/RAF/-MEK/ERK pathways in many circumstances. The biochemistry, biology and regulation of ERKs have been extensively described [1,3].

In keeping with their role in survival and proliferation, the components of RAS/RAF/MEK/ERK signalling are frequently dysregulated in cancer. Aberrant activation of RAS/RAF/MEK/ERK pathways can be driven either by abnormal receptor kinase activation or by oncogenic mutations of the pathway components. KRAS and NRAS are the most common oncogenic mutations in human cancer [6], though HRAS is also involved [7]. BRAF mutations are present above all in melanomas, papillary thyroid carcinomas and colorectal cancers [8].

In this review, we will focus on recent findings on ERK1/2 kinase signalling, their substrates and their role in tumour progression, as well as on the discovery of mutant isoforms and the important role they play in the dysregulation of MAPK signalling responses to therapy. We will also discuss the role played by ERK1/2 mutants in acquired resistance to targeted therapy and radiotherapy that has recently emerged in the literature in studies on MEK inhibitor-based therapy in in vivo and in vitro models.

## 2. ERK1/2 Activation 

The numerous signals that activate MAPK kinases include those from growth factor receptors, integrins, Src and Fyn, and G-protein coupled receptors. ERK1/2 activations occur at both cytosol and plasma membrane and subcellular localization determines the ERK module sensitivity [9]. ERK1/2 activation are also detectable on endo-membranes [10,11]. Since ERK1/2 interacts with the caveolae through caveolin 1, which, with its partner cavin-1, facilitates the recruitment of ERK1/2 to caveolae to balance ERK signalling and other types of signalling, ERK signalling in turn enhances growth and metastasis formation in tumours [12,13,14].

In a simplified model, the classical ERK-MAPK cascade is activated by a tyrosine kinase receptor binding mechanism, followed by autophosphorylation of the cytoplasmic tails of the receptor and the recruitment of Grb-2, which binds the guanine exchange factor, SOS, which in turn interacts with and activates small GTPase RAS [15]. RAF is recruited by the plasma membrane and binds GTP-loaded RAS, which undergoes alterations in the 14-3-3 interaction. This interaction allows conformational changes in the RAF that release its kinase domain for further phosphorylation by kinases (PKC, Src) [16,17]. Activated RAF phosphorylates and activates MEK. MEK is fully activated when it is phosphorylated by RAF and PAK1 [18,19]. Upon activation, RAF mediates phosphorylation of MEK1 and MEK2, which in turn phosphorylate ERK1 and ERK2. ERKs are dually phosphorylated by MEK1 and MEK2 on a TEY sequence corresponding to Thr202 and Tyr204 in ERK1 and to Thr183 and Tyr185 in ERK2. Full ERK activity requires dual phosphorylation, with mutations of either site generating a dominant-negative molecule that inhibits signalling by binding MEKs and downstream targets [3]. Once active, ERK dissociates from MEK, dimerizes and translocates into the nucleus, where it phosphorylates many substrates including transcription factors, whereas in the cytoplasm ERK phosphorylates cytoskeletal proteins and kinases. ERK1/2 phosphorylation of the many substrates with different functions determines the type of responses [20]. The effect of phosphorylation of specific substrates upon the final response is demonstrated, among other things, by the disruption of GAP junction intercellular communication after connexin 43 phosphorylation [21,22] and by cell migration alterations after myosin light chain phosphorylation [23]. Once they have been activated, MAPKs generally act at the level of cytoplasm and nucleus by modulating transcriptional responses within the context of tissue or state of the cells. Elk1, which is the transcription factor that has been studied most, combines with SRF (serum responsive factors) once it has been phosphorylated by ERK1/2 to potentiate ERK1/2 target gene transcription. ERK-mediated phosphorylation of transcription factors, along with the consequent conformational changes, regulates transcription or binding with other factors, increasing or abolishing transcription itself. In some cases, ERKs regulate the activity of their substrates through a protein-protein interaction that is independent of any phosphorylation mechanism but is dependent on the ERK active state. This is the case of ERK2-mediated activation of Topoisomerase II-alpha [24] and of PARP [25]), of ERK1/2-mediated retinoblastoma-lamin A complex disruption [26] and of MKP3 activation by direct interaction with ERK2 [27], and of ERK2 as a transcriptional repressor of interferon signalling [28]. This property is not exclusive to ERKs but may be observed in other MAPKs, such as p38 which are reported to perform a non-catalytic function [29].

## 3. ERK1 and ERK2: Distinct Functions or Functional Redundancy

Although ERK1 and ERK2 are co-expressed in many tissues, the extensively documented greater abundance of ERK2 than of ERK1 could be the first sign of distinct functions. Indeed, functional studies concluding that ERK1 and ERK2 are not redundant relied on the fact that ERK2 null mice are embryonic lethal at E8.5 [30,31], whereas ERK1 null mice generally display a normal phenotype [32,33]. ERK1 and ERK2 have been shown to play different functions in other studies, although the two kinases do not ignore each other at a functional level. Indeed, ERK1 null mice have an increased rate of learning and better long-term memory than wild type mice, which is in line with the notion that ERK signalling is already involved in these processes [34]. Moreover, enhanced ERK2 activation is observed in primary neurons isolated from ERK1 knock-out mice, with no alterations in MEK activation. Authors suggested that ERK1 exerts an inhibitory effect on ERK2. The main conclusion that can be drawn is that the proliferative signal is mediated by ERK2 whereas ERK1 exerts some form of inhibitory function. This conclusion, however, conflicts with new data demonstrating that disruption of ERK2 alleles leads to severe development defects that are fully rescued by the transgenic expression of ERK1 [35]. The increase in ERK2 activation can be a compensatory mechanism to maintain global ERK activity rather than the result of an ERK1-mediated inhibitory function.

ERK2 plays a major role in transmitting the transformation signal whereas ERK1 does not, as recently shown in a study in which Ras-induced epithelial mesenchymal transition (EMT) was found to engage ERK2 but not ERK1 [36]. The authors of this study very recently demonstrated that the mechanism of ERK2-mediated EMT implies the induction of Rac/JNK signalling axis activation via Dock10 (dedicator of cytokinesis 10) [37]. In a different paper they also demonstrated the important role played by ERK2 since the mere overexpression suffices to induce an EMT marker expression similar to that observed upon induction by oncogenic RasG12V. It is noteworthy that ERK2 is required upon oncogenic RasG12V induction of stem-like cell markers [38]. By mapping genome-wide kinase-chromatin interactions, the authors of another study found that, in human embryonic stem cells (hESCs), ERK2 binds chromatin closer to cell-cycle, metabolism and pluripotency-associated genes and that this interaction is mediated by ELK1. The binding of ERK2/ELK1 helps preserve the identity of hESCs, as shown by the fact that genes involved in lineage commitment are repressed in its absence [39].

Taken together, these data suggest that the conclusion that ERK1 and ERK2 play distinct functions is not, however, supported by the recent results of an in-depth analysis based on a large body of data demonstrating the disruption of ERK1 and ERK2 by various technologies, including tissue-specific disruption [35,40] More importantly the transgenic ERKs-deficient studies using a combination of genetic approaches provide evidence of the functionally redundant role of ERK1 and ERK2. Authors tested different combinations of ERK1 and ERK2 alleles and investigated the effects of ERK1/2 different gene dosage then establishing a close correlation between total ERK1/2 activity and developmental defects. They concluded that the different gene dosage-dependent mechanism of ERK1/2 signalling contributes to global ERK1 and ERK2 activity and overexpression of ERK1 rescues all defects of ERK2 deficient mice suggesting that ERK1 replace ERK2 functions.

Nevertheless, the role of ERK1 and ERK2 in a tumour context it is worth examining more closely. As a matter of fact, comparing ERK2 with ERK1 in breast cancer cells invasiveness, ERK2, not ERK1, drives cells invasiveness migration by reducing the expression of motility suppressors genes [41]. This result implicates ERK2 rather than ERK1 in the transformation mechanism.

More in-depth studies are necessary to definitely demonstrate the functionally redundant role of ERK1 and ERK2, and whether ERK2 depends on specific contest.

## 4. ERK1/2 Substrates

Early attempts to discover ERK1/2 targets made use of mass spectromics-based techniques. These studies were based on the use of the ERK signalling activator (EGF), pathway-specific inhibitors, or a cell system engineered to induce RAF/MEK/ERK pathways by stimulating cells in an oestrogen-dependent manner [42]. The large number of ERK downstream molecules is a sign of how many functions are performed by ERK1/2. The authors compiled a tentative list per category: Transcription Factors, Kinases and Phosphatases, Cytoskeletal Proteins, Signalling Proteins, Apoptotic Proteins, Proteinases, and other proteins that do not fall into any of those categories [3,20,43].

Biochemical and genetic approaches helped identify numerous ERK1/2 substrates, though with the advent of phospho-proteomic techniques, the number of ERK substrates has increased and researchers have assembled ERK target phospho-sites in an archive that is accessible online [20].

The substrate targets of ERK1/2 include Myc, which has been described in RAS overexpressing cells. The level of Myc is regulated by two kinases: ERK phosphorylates Ser62 and stabilizes Myc, thereby enabling the latter to act at the promoters of growth and transformation genes, whereas GSK3-beta phosphorylates Myc at Thr58, thereby promoting the ubiquitination and degradation of Myc after protein complex formation and Ser62 dephosphorylation [44]. RAS, activating ERK and PI3k/AKT which inhibits GSK3-beta, induces the phosphorylation of Ser62 and maintains Thr58 in an unphosphorylated form. Thus, RAS activation or mutation in tumour cells can effectively stabilize Myc. Mutations in Thr58, which are frequent in some lymphomas such as Burkitt’s lymphoma, result in a lack of degradation and Myc stabilization [45]. Within this scenario, ERKs are involved in maintaining the oncogenic phenotype.

An example of the role of ERK as an oncogenic phenotype keeper has recently been documented in a system that commonly harbours mutations in NRAS, HRAS and KRAS, i.e., embryonal rhabdomyosarcoma in vivo and in vitro [46]. In their study, the authors performed a high-throughput drug screen to discover compounds that selectively target the MAPK pathway and viability of these cells. Trametinib (non-ATP competitive allosteric MEK1/2 inhibitor) was the MEK inhibitor that most effectively reduced ERK activity and cell viability in these cells.

By analogy with reports from studies on embryonic stem (ES) cells in which ERK2 affects gene expression by impacting chromatin [39], ERK2 has been hypothesized to act similarly by blocking the expression of the pro-differentiation gene Myogenin while maintaining the oncogenic phenotype. These findings support a model in which ERK2 phosphorylates and stalls RNA Pol II at the Myogenin promoter in RAS-mutated rhabdomyosarcoma, thereby inhibiting gene expression. MEK/ERK inhibition by the MEK inhibitor trametinib caused ERK2 loss at the Myogenin promoter and the release of Myogenin transcription stalling. Myogenin-mediated chromatin remodelling then enables the transcription of genes required for late myogenesis.

By contrast, MEK inhibition induced Myc down-regulation, thereby ensuring the interruption of the growth gene transcription and the reversal of myogenesis inhibition [47,48,49]. Interestingly, since in vivo tumour growth suppression was incomplete, remission was achieved by combining trametinib with the IGF1R inhibitor [46]. The authors highlighted the role played by ERK2 in blocking differentiative transcription and maintaining the transformed phenotype by stabilizing Myc. This confirms that ERK2 is, at least in rhabdomyosarcoma, the upstream kinase of Myc, as we had previously observed [50]. The data from this paper also reveal that ERK2 interacts with chromatin, thereby representing an alternative mechanism to catalytic activity.

## 5. ERK1/2 Mutants

The ERK1/2 pathway impairment observed in cancer very frequently depends on genetic alterations that affect the MAPK upstream components such as RAS the most common oncogene with 30% of incidence and RAF which is very frequent in melanoma [51]. Nevertheless, ERKs mutants have recently entered the spectrum of mutations believed to be responsible for MAPK dysregulation and drug resistance [52].

Studies of the biochemical, biological and pathological functions of proteins make use of constitutively active mutants that acquire the ability to perform an intrinsic activity while evading regulation. Although the use of mutants would have allowed the ERK1 or ERK2 pathway to be dissected, the particular mode of MAPK activation and the lack of suitable technologies meant that such mutants did not become available until relatively recently.

Owing above all to the rarity of ERK mutations, the functional impact of ERK mutations in diseases was once almost unknown and poorly understood. More recently, with the advent of a new and more comprehensive genome sequence analysis, not only have mutations of many unknown cancer genes or mutations of genes not yet related to specific cell functions been characterized [53,54,55], but it has also been possible to clone new mutant genes and thus perform functional studies.

In order to study the interaction between MAP kinase components and their specificity in signalling transduction, constitutively active, gain of function and dominant negative mutants were created. As an example, an MAPK kinase MEK1 mutant was used to investigate the impact of MEK/ERK signalling in transformation and differentiation [56].

The authors of some studies searched for ERK1 and ERK2 mutants to investigate whether ERK mutation might be able to confer resistance to RAF and MEK inhibitors and to induce MAPK reactivation under MEK inhibitor therapy. Thereafter, ERK2 mutants were identified as rare cancer-associated gain- and loss-of-function gene products [52]. The gain-of-function ERK2 isoforms comprise a class of proteins whose response to RAF, MEK and ERK inhibitor signalling varies. Between the identified ERK2 mutants the ERK2D^321N^ and ERK2E^322K^ has been found in human tumour and cancer cell lines [57,58]. The discovery of these ERK2 mutants and their responsiveness to specific inhibitors is likely to provide useful indications for new therapeutic strategies for patients harbouring these mutations. When other authors used a random mutagenesis screen in a previous paper, they identified multiple point mutations in ERK1 and ERK2 that were tested to determine whether they confer resistance to ERK or RAF/MEK inhibitors [58]. Interestingly, the authors discovered that ERK inhibitor resistance implied responsiveness to RAF/MEK inhibitors and, vice versa, responsiveness to ERK inhibitors implied RAF/MEK inhibitor resistance. This led to an alternating ERK and RAF/MEK inhibitor treatment regimen designed to avoid the development of resistance to these agents. One of the mutations of ERK2 that these authors detected in their screen, MAPK1^E322K^, was identified in cervical and head and neck carcinoma [59,60]. The authors of this study showed that MAPK1^E322K^ did not exhibit increased basal kinase activity in vitro but the kinase remained activated in vitro owing to reduced DUSP (dual-specificity phosphatase) binding and loss of negative regulation conferring resistance to RAF/MEK inhibitors. In human cancer cell lines, a mutation in codon 322 of the extracellular signal-regulated kinase (ERK2^E322K^) exhibits constitutive phosphorylation and the protein presents an oncogenic potential proved also by loss of anchorage-dependent growth. Indeed, in transgenic flies carrying the corresponding ERK2 mutation, alteration in eye development occurred [61,62].

As the development of drug resistance is one of the main problems encountered in targeted therapy and radiotherapy, efforts have been made to identify ERK1/2 mutations that confer resistance to RAF/MEK or ERK1/2 inhibition and to radiotherapy.

Point mutations in ERK2 had previously been identified in conserved residues L73P and S151D and had been shown to confer 8- and 12-fold increases in ERK2 specific activity. When combined, these two mutations play a synergistic action that results in a 50-fold increase in ERK2 activity [63]. The authors of that study showed that mutants are only partially sensitive to MEK inhibitor U0126. This finding further highlights the frequently insufficient effects of the MEK inhibitor in abolishing ERK activity and the need to look for an effective and specific ERK inhibitor. In another study, mutations in the “gatekeeper” residues, which confer selectivity for nucleotide and small molecule inhibitor binding, were found to regulate autoactivation of ERK2 through an intramolecular mechanism [64]. That study helped to establish that the “gatekeeper” and close domains form a unit that maintains ERK2 in an inactive state in the absence of any MEK1/2 signal and that its mutation disrupts regulation by restraining ERK2 autoactivation.

The most frequent response to clinical therapies is acquired resistance to drugs due to the reactivation of Raf/MEK/ERK pathways, a mechanism that remains elusive. Thus, the use of small molecules designed to specifically target ERK1/2 became highly recommended, particularly for tumours that depend on RAF/MEK/ERK dysregulation.

ERK mutants can also develop during ERK inhibitor treatment, as was recently discovered using ERK-inhibitor-sensitive cancer cell lines [65] (see below).

## 6. MEK/ERK Inhibition and ERK Reactivation 

Since the RAF/MEK/ERK pathways play a central role in many cell functions of normal and diseased tissues, the balance between active and inactive states of the cascade must be tightly regulated. In this regard, a number of regulators, including the small GTPases RAS and Rap, phosphatases, scaffolds, coordinate RAF/MEK/ERK signalling by affecting the magnitude, duration and compartmentalization of the pathway activities [1,66,67].

Interestingly, the activation state of MEK1/2 is not affected exclusively at the phosphorylation level but also results from changes in the cellular levels of kinases: RAF/MEK/ERK can feedback up-regulate MEK1 at the transcriptional level and feedback down-regulate MEK2 at a post-translational level that is sensitive to the proteasome activity [68]. This mechanism, which was the first demonstration of feedback regulation at the cellular level of MEK1 and MEK2, might have a significant impact on development and on cancer. It has also been demonstrated that adhesion through PAK-induced MEK1 phosphorylation regulates the MEK1-ERK interaction whereas adhesion-induced ERK phosphorylation of MEK1 is inhibitory [69]. This is an ERK2 feedback phosphorylation mechanism that controls MEK1 complex formation and activation.

The non-oncogenic MAPK cascade during normal growth factor signalling is finely regulated so as to ensure that ERK activation and de-activation meet the requirements of transcriptional and translational events for cell growth and morphology [70]. Experimental evidence has shown that positive and negative feedback plays a critical role in the maintenance of balance sensitivity and cellular homeostasis [71].

By contrast, the controlled baseline sensitivity in the dysregulated MAPK cascade is lost as a result of chronic high ERK activity that persistently stimulates transcription and translation in order to maintain aberrant growth [72].

Since the discovery of genetic mutations in the components of the RAS/RAF/MEK/ERK pathway and of dysregulation of the pathway itself, intense efforts have been made to develop and test compounds that target MAPK pathway components.

Although the use of BRAF inhibitor to target MAPK-dysregulated tumours was found to be a promising therapy [73,74], the response duration was limited owing mainly to the reactivation of MAPK by multiple mechanisms, as observed in melanoma, a typical BRAF-mutated tumour. [75]. Selective MEK inhibitors were shown to yield promising results, though the benefit of this therapy was limited by drug resistance, a problem that is shared by inhibitors of other oncogenic kinases. Multiple potential mechanisms of acquired resistance, some of which were observed in clinical studies, were identified. Since the main drawback of these therapies is ERK reactivation, in this paragraph we are going to preferentially discuss mechanisms that are dependent on ERK signalling respect to the others. Targeted therapies often fail when mutations or amplifications of drug targets or alterations in downstream pathways concomitantly occur thus evading the activity of the drugs themselves.

In this regard, some of the MEK1 mutations identified markedly attenuated the ability of MEK1 inhibitor to prevent ERK1/2 activation, while others induced BRAF inhibitor cross-resistance [76] or were detected in a post-relapse biopsy in patients with acquired BRAF inhibitor resistance, thus suggesting that MEK1 mutation may underlie acquired resistance to BRAF inhibitors.

Until recently, secondary BRAF mutations such as to cause BRAF and MEK inhibitor resistance could not be detected, whereas the selective amplification of mutant BRAF was detected in colorectal cancer cell lines that are resistant to the MEK inhibitor [77] as a result of an underlying mechanism of MEK1 hyperactivation. However, whole exome sequencing recently led to the detection of a secondary mutation in mutant BRAF, which confers resistance to RAF inhibitor [78], in a brain tumour that was progressing though not before the tumour was treated. A secondary BRAF resistant mutation was also detected in metastatic BRAF mutant melanoma [79] using detailed imaging studies and genetic analyses. In addition to BRAF mutations, BRAF selective amplification was found to be the mechanism of acquired MEK inhibitor resistance by two groups in a colorectal cancer cell line in vitro [77,80], with MEK hyperactivation being observed in these resistant cells. Moreover, CRAF hyperactivity was also identified as the mechanism underlying the switching of tumour cells from BRAF to CRAF dependency in preclinical studies. Taken together, these data indicate that resistance to BRAF and MEK inhibitors largely depends on the reactivation of ERK, which restores growth and survival, particularly in the melanoma system.

**Figure 1:**
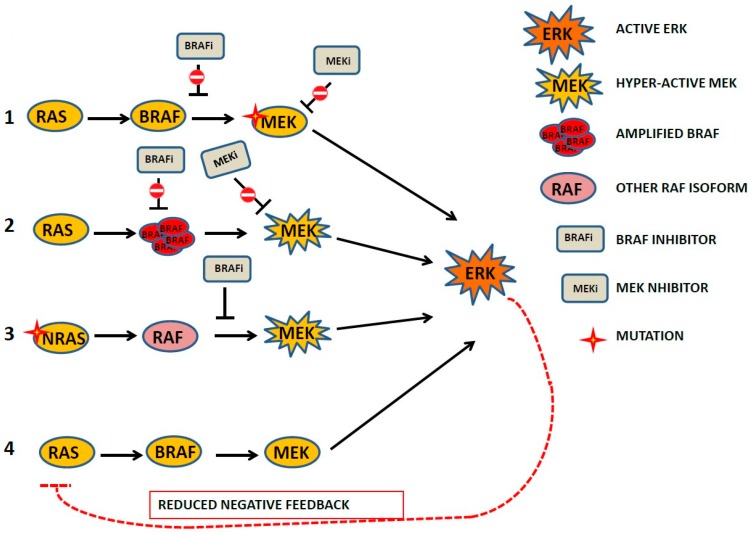
Synthetic representation of the main mechanisms leading to BRAF and MEK inhibitors acquired resistance frequently present in melanoma and colorectal cancer in vivo and in vitro. (1) MEK1 mutation which attenuated the ability of MEK inhibitor to prevent extracellular regulated kinases (ERK) activation; some mutation leads to cross resistance to BRAF inhibition; (2) amplification of BRAF leads to acquired resistance to MEK Inhibitor and to BRAF inhibitor depending on hyper-activation of MEK ; (3) NRAS mutant (Q61R) was identified in a progression phase, resistant cells in vitro maintained both phospho-active MEK and ERK despite the presence of BRAF inhibitor; (4) reduction of ERK-dependent negative feedback on the mitogen-activated protein kinases (MAPK) pathway.

In particular, KRAS and NRAS mutant tumours treated with MEK inhibitor are strongly but transiently responsive to the inhibitor and more susceptible to MAPK pathway reactivation [81]. CRAF, BRAF and ERK2 are the main kinases underlying the responsiveness to MEK inhibitor [82]. ERK reactivation under MEK inhibition has been reported to be the main problem in therapeutic strategies aimed at NRAS and KRAS mutant tumours [83], with ERK being the critical node between responsiveness to MEK inhibition and MAPK reactivation. It is for this reason that ERK inhibitor combined with MEK inhibitor is one of the new strategies being used to achieve more effective and persistent MAPK pathway inhibition. The synthesis of the main ERK-dependent mechanisms of acquired resistance to MAPK inhibitor (BRAFi, MEKi) identified in melanoma and colorectal cancer is shown in Figure 1.

Non-ERK dependent mechanisms of resistance that do not rely on ERK signalling were described, as were other ERK independent pathways that sustain tumorigenicity of BRAF mutant tumours such as melanoma. Microarray gene expression profiles in resistant cells revealed the overexpression of RTKs, including PDGFR, KIT, MET and EGFR, when compared with parental cells. The fact that IGF1R hyper-activation, which has also been identified as an ERK-independent mechanism, involves other signalling pathways such as PI3K/AKT suggests that the co-targeting of IGF1R/PI3K and MEK may be considered as a promising therapy to restore apoptotic response in ERK-independent resistant tumours [84].

## 7. Impact of Mutant ERK1/2 on Anticancer Therapy Outcome

The development of resistance is a problem that is common to classical chemotherapy and targeted therapy. Indeed, despite the advances that have been in chemotherapy over the last 50 years, most patients still have relapses after responding well at the beginning of treatment, owing to acquired resistance.

Cancer drug resistance is a complex mechanism that cannot be discussed in depth in this review. We will thus limit our discussion to MEK inhibitor acquired resistance rather than address the failure of other types of chemotherapy.

Just as an overuse of antibiotic leads to the selection of mutated, resistant bacteria strains, when tumour cells that are highly unstable owing to constant proliferation receive a lethal dose of inhibitor, they develop mutations that help them to survive and acquire resistance to the inhibitor. The MAPK pathway, which is often deregulated or mutated, is a therapeutic target for many human cancers. Indeed, MAPK reactivation, which often occurs following inhibition of upstream kinases, has become one of the main challenges in therapy design.

In 2006, Solit and co-workers used small molecule inhibitors of MEK and an integrated pharmacological analysis to show that mutant BRAF predicts sensitivity to MEK inhibition [73]. In addition, they showed that since melanoma-carrying BRAF mutations are reliant on MEK-ERK signalling to a far greater extent than RAS mutants, the former tumours are highly sensitive to pharmacological MEK inhibition.

Extensive studies in vitro as well as in patients have demonstrated that MAPK inhibition in BRAF^V600E^ mutant metastatic melanoma is accompanied by drug resistance and the recovery of ERK activity, and that the conditions of patients treated with BRAF and MEK inhibitor progress within months [85]. By contrast, the selective knocking down of ERK1 or ERK2 in another study killed melanoma cells and enhanced the action of the BRAF Inhibitor [86]. Multiple complex and context-dependent mechanisms confer resistance to the BRAF inhibitor in melanoma. Indeed, when biochemical analyses were performed on BRAF resistant melanoma cell lines generated in some studies, they revealed MEK/ERK reactivation through RAS, which is the key resistance mechanism in these cells. The authors of those studies also found that FGFR3 enhanced activation, thereby contributing to MAPK pathway recovery since inhibition of the FGFR3/RAS axis restored the responsiveness to BRAF inhibition [87]. Since the most common clinical resistance mechanism implies the reactivation of the RAF/MEK/ERK pathway by various mechanisms, the targeting of ERK itself has become an emerging novel therapy in MAPK kinase-driven cancers and ERK inhibitors are currently being tested in clinical trials. To demonstrate the impact of ERK in the recovery of the MAPK pathway, a random mutagenesis screen was used to identify ERK1 and ERK2 point mutations that might confer resistance to ERK or RAF/MEK Inhibitors [58]. Interestingly, the mutation resistant to the ERK inhibitor recovered sensitivity to the RAF inhibitor and vice versa, thus suggesting a regimen of alternating inhibitors to elude resistance to these drugs. Moreover, ERK2 is recurrently mutated in head, neck and cervical cancers [59,60]. In 2016, when Brenan and co-workers used saturation mutagenesis to comprehensively characterize the activity and drug sensitivity of a wide range of ERK2 mutants, they identified a rare ERK2 mutant that is found in human tumours and is associated with gain- or loss-of-function activity (GOF, LOF) and a differential responsiveness to MAPK-directed therapies [52]. The authors of that study generated an ERK2 mutant library to characterize ERK2 mutant responses to therapies that were subsequently to be used for genome-guided oncology. These studies will provide a basic structure for current and future classifications of disease-associated mutants.

A classic example of the impact of ERK mutants on the outcome of therapy emerges from a recent report on the effects of ERK2 on target mutation and amplification in which the ability of the specific ERK inhibitor compound to bind mutant ERK was found to be impaired [65]. Moreover, studies based on models of ERK inhibitor resistance mechanisms found that MEK, ERBB receptor and PI3K/mTOR pathway inhibitors were effective in overcoming the acquired ERK inhibitor resistance. These results are similar to those obtained by other authors that used a different ERK inhibitor that induced acquired resistance attributable to an ERK1 mutation deficit in inhibitor binding. Since ERK1-mutant cells retain MEK inhibitor sensitivity, they proposed MEK/ERK or BRAF/ERK inhibitors as alternative treatments [88].

Taken together, these data suggest that combinations of different inhibitors may serve as a paradigm for alternative treatments that can be tailored to specific tumours.

## 8. The Role of ERK1/2 Signalling in Cancer Cell Radioresistance

Radiation therapy (RT), which is one of the most common cancer-related treatments, is used in approximately 50% of all cancer patients and accounts for around 40% of the curative treatment effect [89,90,91]. RT kills cancer cells by using ionizing radiation (IR); however, cancer cell resistance to IR frequently causes local recurrences and metastases [92,93,94]. Thus, understanding the molecular mechanisms responsible for radioresistance is crucial for improvements in RT to be made. IR removes cancer cells by inducing direct or highly reactive oxygen species (ROS)-mediated DNA damage, particularly DNA double-strand breaks (DSBs), the most lethal form of damage, which in turn leads to apoptosis-, necrosis-, senescence- or autophagy-mediated cancer cell death [95,96]. Furthermore, IR promotes cancer cell death by activating the host anti-tumour immunity [97].

Although RT-induced anti-cancer toxicity starts promptly and continues for weeks to months after the end of treatment, cancer cells may evade IR-induced cell death through a number of different mechanisms [95]. Indeed, the success of RT is determined by the ability of treatment i) to induce irreparable DNA damages; ii) to redistribute the surviving cells into the G2/M cell cycle phase, which is the most radiosensitive phase; iii) to prevent the surviving cells from repopulating the death fraction; and iv) to promote the re-oxygenation of hypoxic tumour areas. Thus, cancer cells can determine RT failure by promoting DNA repair and counteracting their redistribution, repopulation and reoxygenation [97]. These features are usually referred to as the “four Rs of radiobiology”, while recent evidence suggests that the intrinsic radioresistance of cancer stem cells (CSCs) is the fifth “R” [98,99,100,101].

IR has been shown to activate ERKs, which sustain cancer cell radioresistance by regulating the five “Rs” [93,102,103,104,105,106,107,108,109,110,111,112]. Following radiation, ERK1/2 is activated through dual tyrosine and threonine phosphorylation by MEK1/2 and the activation, in turn, leads to the phosphorylation/activation of over 160 substrates [113]. While the exact mechanisms responsible for the activation of MEK1/2-ERK1/2 signalling by radiation has not yet been clearly elucidated, the rapid activation of HER family receptors following ionizing radiation has been shown to play a key role [114].

**Figure 2:**
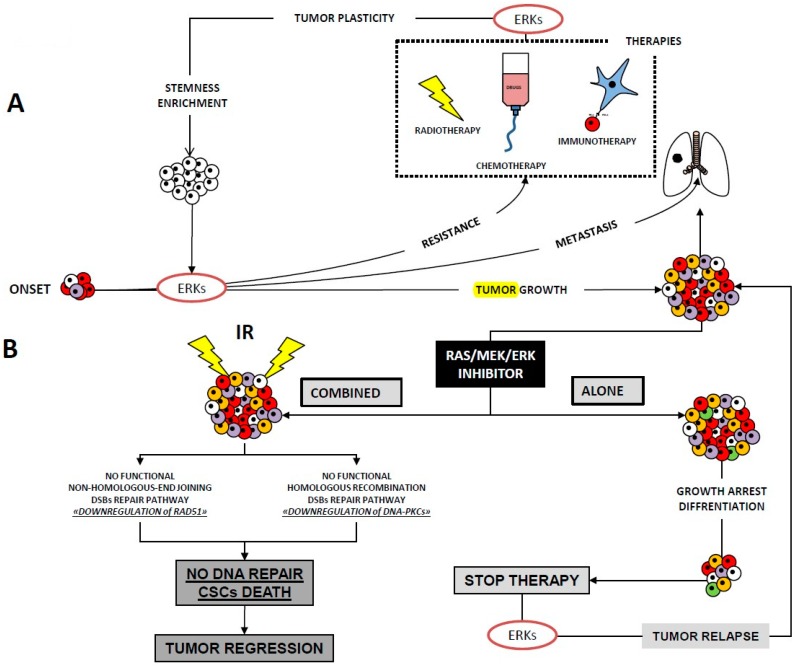
Schematic representation of ERK1/2 signalling leading to multiple tumour responses in: (**A**) onset of tumour growth, resistance to chemo- and radiotherapy (dotted square), metastasis and tumour plasticity due to ERK-induced radiotherapy, chemotherapy or immunotherapy, often leading to selection of cancer stem cell (CSCs); (**B**) RAS/MEK/ERK inhibitor alone has limited benefit whereas followed by irradiation (IR) inhibits the mechanism of DNA repair and favours CSCs death with tumour regression.

Furthermore, studies have demonstrated that radiation-induced G2/M checkpoint response requires ERK1/2 signalling activation [115]. ERK signalling modulates the repair of damaged DNA by sustaining the activity of the non-homologous end-joining pathway (NHEJ), which is mediated by ataxia-telangiectasia-mutated (ATM) and ataxia-telangiectasia related (ATR) protein, as well as that of the homologous recombination pathway (HR), which is mediated by DNA-PKCs [116,117,118,119,120,121]. In keeping with their key roles in DNA repair, ATM, ATR and DNA-PKCs have been shown to determine the activation of ERKs induced by IR [115,122], which in turn facilitates the activation of ATM, ATR and DNA-PKCs [116,123] and triggers a self-maintaining pro-DNA-repair molecular loop. Interestingly, the impact of ERK pathway activation in IR has been found to be cell type-dependent. It generally promotes cell cycle arrest and cell death in normal mammalian cells [115] but survival in many types of cancer cells [107,111,112,116,124,125,126,127,128], by inducing the transcription of genes involved in DNA repair in both cases [115,122,129,130,131]. Given the key role played by ERKs in the regulation of the cell cycle [132], this signalling has been widely investigated on account of its ability to participate in the redistribution of the cell cycle phases induced by IR. Taken together, the evidence collected shows that the ERK activation induced by IR allows cancer cells to overcome cell growth arrest in G2/M, which is the most radiosensitive cell cycle phase, thereby causing radioresistance [133,134,135,136,137,138]. Bearing in mind that IR-induced ERK activation occurs constantly regardless of the cancer cell type and that the therapeutic efficacy of RT largely depends on G2/M arrested cells, the ERK-mediated mechanism of G2/M escape must be considered as a fundamental element when drawing up strategies aimed at radiosensitizing cancer cells. The schematic representation of ERK1/2 signalling leading to multiple tumour responses is shown in Figure 2.

Our group has previously reported that the radiosensitivity of cancer cells increases and that tumour growth is severely compromised following MEK/ERK inhibition after IR in cell systems from RAS mutant tumour cell lines and non-RAS mutant cell lines, the embryonal rhabdomyosarcoma, prostate-derived cancer cells and gynaecological-derived cell lines [107,111,112], while other groups have reported similar findings in different systems [126,139]. Cells deprived of ERK activity lack the downstream ERK targets responsible for cell survival, for DNA damage repair and for oncogenic phenotype expression, as occurs in Myc, whose silencing radiosensitizes prostate cancer cells [107]

The repopulation of a dead fraction by cancer cells is a crucial event in radioresistance and relapse [140]. Whether the ability to repopulate the dead fraction is strictly related to DNA-repair and redistribution and the mechanisms underlying this phenomenon is as yet unclear. Nevertheless, one possible mechanism may consist in proliferation signals reaching non-irradiated and radioresistant tumour cells from lethally irradiated tumour cells through the ERK-dependent activation of caspase 3, which in turn triggers growth-stimulating signals [132,141,142,143]. Particularly, it has been shown that the caspase-3 mediates the activation of phospholipases A2, that in turn generates prostaglandin E2—known to promote tumour repopulation, through a pathway called “Phoenix Rising” [141]. Furthermore, other studies have shown that the recruitment of macrophages that governs elimination of apoptotic cells after radiotherapy induces tumour repopulation by production of a clearance-related cytokine milieu including PGE2 [144].

Hypoxia, which is one of the most important parameters of radioresistance, reduces the process of IR-induced “oxygen fixation” that eventually leads to cell death by increasing ROS accumulation. Indeed, cells irradiated in hypoxic conditions are 2-3 times more resistant to IR [102,103]. Hypoxia in cancer cells usually activates a complex cell signalling network that includes the ERK pathway [145]. ERK phosphorylates/activates the hypoxia inducible factor-1 (HIF-1), a master transactivator in hypoxic conditions [146] that in turn regulates the transcription of hypoxia-adaptive proteins, which, by promoting survival under low oxygen levels, maintains hypoxia by creating chaotic and malfunctioning blood vessels [147,148] and eventually promote radioresistance [110,149].

Furthermore, hypoxia has been shown to preserve and enrich cancer stem cell (CSC) populations, which are the cornerstone of radioresistance [102]. CSCs are a small subpopulation of cells within tumours that develop capabilities of self-renewal, differentiation and tumourigenicity when transplanted into an animal host [100]. CSCs are not only able to maintain themselves but also to differentiate, thereby establishing heterogeneity in the bulk tumour. If compared with bulk cancer cells, CSCs possess an elevated and more efficient DRR (deficiency in DNA damage responses), which contributes to radioresistance [101,102,103]. Furthermore, while the acquisition of CSC traits is classically considered a stochastic process, recent studies have shown that this process is not random and can be induced by injury of non-CSCs, as observed after RT [104,105]. This process, which is called “cancer cell plasticity”, can result in CSCs becoming refractory to treatment, and the treatment itself can promote the CSC phenotype, thus determining the failure of the cure [106]. CSCs thus represent a cornerstone that must be eradicated if the cancer is to be cured [101,102,103] and treatments should aim to restrain IR-induced reprogramming in order to improve the efficacy of RT [106]. The signalling pathways in CSC biology are increasingly being used to investigate the mechanisms underlying therapy resistance [94,150,151]. ERKs have been shown to be vital for CSC tumourigenicity [108] and for RT-induced CSC selection and accumulation, and ERK inhibition impairs the cancer stem cell population by restoring the radiosensitization of cancer stem cells [152,153,154,155]. Thus, the evidence available to date indicates that inhibiting the ERK signal transduction pathway is a promising means of enhancing the radiosensitivity of tumours.

Interestingly, ERKs amplification or mutations have been shown to play a key role in the acquisition of resistance to treatments which lastly causes recurrence, cancer dissemination and death [94,156]. Resistance to cancer drugs can be classified as either pre-existing or acquired. Pre-existing (intrinsic) resistance describes the case in which the treatment selects a subpopulation resistant to therapy. The growth of this resistant population makes the therapy ineffective. On the other hand, acquired therapy resistance broadly describes the case in which resistance develops during the course of therapy from a population of cells that were initially sensitive [157]. Considering that mutations of ERKs have been identified in highly radioresistant cancer it is unlikely that they occur as an effect of radiation. However, we reported that radiation combined with MEK/ERK inhibitor treatment in in vivo and in vitro system of rhabdomyosarcoma and prostate cancer cells works better owing to MEK/ERK inhibitor-mediated down regulation of components of DNA repair machinery [107,112].

The knowledge that ERK1/2 mutations severely compromise targeted therapies [65] is the results of several investigations aiming to isolate ERK1/2 mutants helpful in functional studies for the identification of drug resistant mutants and ultimately, when relapse occurs, guide the choice of therapy. Investigations using ERKs mutant-engineered cells to be used in radiation experiment might be of help to better clarify the role of ERK1/2 in radiation responses and to characterize the combined modality treatment that involves MAPK pathway inhibitors and radiation.

## 9. Concluding Remarks and Future Perspectives

The field of research on the ERK signalling pathway and cancer continues to expand. Early studies focused on aberrant MAPK pathways in cancer, with attention being paid above all to mutations in the upstream components of the MAPK pathway, RAF, RAS and MEKs, though the particular dual phosphorylation modality activity of ERKs meant that any ERK functional mutations were unlikely. Nevertheless, the need for more successful and durable targets in MEK inhibition therapy, in which MAPK pathway reactivation occurs early, led to the development of novel approaches aimed at identifying ERK mutants and at guiding therapeutic strategies designed to radically modify treatment in patients. Saturation mutagenesis approaches helped to identify ERK1 and ERK2 mutants, which were in some cases found in human cancer. Studies on the ERK2 mutant, in particular, revealed that gain-of-function ERK2 mutants are two classes that respond differently to RAF, MEK and ERK inhibitors. These findings might help to design therapeutic strategies that can be tailored to patients whose tumours harbour one of these mutants. In addition, since findings show that though the efficacy of RAF and RAS inhibitors at beginning of treatment ERKs is reactivated, the reasons underlying the inhibition in the final stage of the cascade must investigated and clarified.

All the data in this review indicate that ERK2, the essential effector of RAS and RAF, is the most versatile and functional ERK on account of its ability to affect transcription and bind chromatin as well as other proteins in addition to its classical kinase activity. Studies aimed at the identification of ERK2 mutants are thus particularly useful as a means of identifying ERK inhibitors that may lend themselves to functions that are markedly different from those exerted by the classical ERK2 kinases [52]. To sum up, these approaches may be considered as a first step toward comprehensive genome-guided oncology and be used to draw up a disease-associated mutant classification.

These studies are of fundamental importance in the development of more effective therapies based on a combination of drugs and radiotherapy that are needed to target destructive tumours.

## Abbreviations

ERK	extracellular regulated kinase
MAPK	mitogen-activated protein kinase
MEK	component of MAPK core
PARP	poly (ADP.ribose)polymerase
JNK	c.Jun N-terminal kinase
